# Biodegradable nanoparticles of methoxy poly(ethylene glycol)-*b*-poly( d, l-lactide)/methoxy poly(ethylene glycol)- *b*-poly(ϵ-caprolactone) blends for drug delivery

**DOI:** 10.1186/1556-276X-7-271

**Published:** 2012-05-30

**Authors:** Yodthong Baimark, Yaowalak Srisuwan

**Affiliations:** 1 Department of Chemistry and Center of Excellence for Innovation in Chemistry, Faculty of Science, Mahasarakham University, Mahasarakham, 44150, Thailand

**Keywords:** Biodegradable polymers, Polymer blends, Diblock copolymers, Nanoparticles, Nanoprecipitation method, Controlled release

## Abstract

The effects of blend weight ratio and polyester block length of methoxy poly(ethylene glycol)-*b*-poly( d, l-lactide) (MPEG- *b*-PDLL)/methoxy poly(ethylene glycol)- *b*-poly(ϵ-caprolactone) (MPEG- *b*-PCL) blends on nanoparticle characteristics and drug release behaviors were evaluated. The blend nanoparticles were prepared by nanoprecipitation method for controlled release of a poorly water-soluble model drug, indomethacin. The drug-loaded nanoparticles were nearly spherical in shape. The particle size and drug loading efficiency slightly decreased with increasing MPEG- *b*-PCL blend weight ratio. Two distinct thermal decomposition steps from thermogravimetric analysis suggested different blend weight ratios. Thermal transition changes from differential scanning calorimetry revealed miscible blending between MPEG- *b*-PDLL and MPEG- *b*-PCL in an amorphous phase. An *in vitro* drug release study demonstrated that the drug release behaviors depended upon the PDLL block length and the blend weight ratios but not on PCL block length.

## Background

In the past few decades, biodegradable and biocompatible nanoparticles of methoxy poly(ethylene glycol)-*b*-poly( dl-lactide) (MPEG- *b*-PDLL) and methoxy poly(ethylene glycol)- *b*-poly(ϵ-caprolactone) (MPEG- *b*-PCL) amphiphilic diblock copolymers have shown potential as controlled-release drug delivery carriers because of the small size of their nanoparticles, which improves circulation time in the body and decreases the administration frequency when compared to microparticles which are rapidly cleared by the reticuloendothelial tissue [1,2]. Moreover, for MPEG-*b*-PDLL and MPEG- *b*-PCL nanoparticles suspended in water, PDLL and PCL hydrophobic cores are surrounded by hydrophilic MPEG blocks on the nanoparticle surface to solubilize hydrophobic drugs; this increases blood circulation time and decreases uptake by the liver of the nanoparticles [3-6]. The need for surfactants when preparing nanoparticles of amphiphilic diblock copolymer by the nanoprecipitation method can be removed. The protective effect of the hydrophilic MPEG block is adequate for preventing nanoparticle aggregation. The poly(vinyl alcohol), Span series, Tween series, poly(ethylene oxide) (PEO), and poloxamer (PEO-poly(propylene oxide) block copolymer) have been used as surfactants to stabilize emulsion droplets [7]. These surfactants remain at the particle surface and are difficult to remove which affect the biodegradability and drug release profile of the drug-loaded particles. Also, these remaining surfactants can influence the human body, for example, causing an allergy-like reaction.

In previous studies, much attention was paid to the drug-loaded nanoparticles of these amphiphilic diblock copolymers with different types of chemical compositions and lengths of polymer blocks used [8-11]. Drug release profiles of the nanoparticles depended upon these factors. The physical blending of polymers is an alternative method that has been widely used to adjust the properties of biodegradable polyesters [12-15]. Thus unique properties of polymer blends, quite different from the origin polymers, were obtained. The drug release rate from poly(l-lactide)/PCL blend nanoparticles prepared from the nanoprecipitation method using poloxamer 188 as the surfactant has been adjusted by varying the blend weight ratios [16].

The characteristics and drug release behaviors of the MPEG-*b*-PCL nanoparticles containing drug prepared by the nanoprecipitation method can be controlled by adjusting the processing parameters, such as the organic/water phase volume ratio, polymer concentration, drug/polymer weight ratio, and stirring speed [17]. However, the effect of MPEG-*b*-PDLL/MPEG- *b*-PCL blending on characteristics and drug release of the nanoparticles has not been reported.

In this study, surfactant-free MPEG-*b*-PDLL/MPEG- *b*-PCL blend nanoparticles containing a model drug were prepared by the nanoprecipitation method. Indomethacin was selected as the model drug because of its poor water solubility. The effects of different polyester block lengths and blend weight ratios on the nanoparticle characteristics, drug loading efficiency, and drug release profiles were studied and discussed.

## Methods

### Materials

MPEG-*b*-PDLL and MPEG- *b*-PCL diblock copolymers were synthesized by ring-opening polymerization in bulk under a nitrogen atmosphere as described in the previous works [18,19]. MPEG with a molecular weight of 5,000 g/mol (Fluka Chemical Corporation, St. Louis, Milwaukee, WI, USA) and stannous octoate (95%, Sigma-Aldrich Corporation, St. Louis, MO, USA) were used as the initiating system. Stannous octoate concentration was kept constant at 0.02 mol%. MPEG-*b*-PDLL and MPEG- *b*-PCL with theoretical PDLL/PCL block lengths of 30,000 (30 K) and 60,000 (60 K) g/mol were called MPEG- *b*-PDLL30K, MPEG- *b*-PDLL60K, MPEG- *b*-PCL30K, and MPEG- *b*-PCL60K, respectively. The resulting number-average molecular weights ( *M*_n_) and molecular weight distribution (*MWD*) of diblock copolymers are summarized in Table 1. Indomethacin model drug (99%, Sigma-Aldrich Corporation, St. Louis, MO, USA) was used without further purification. All reagents used were analytical grade.

**Table 1:** Molecular weight characteristics of diblock copolymers

**Diblock copolymer**	***M***_**n, theoretical**_** (g/mol)**^**a**^	***M***_**n, GPC**_** (g/mol)**^b^	***MWD***^**b**^
MPEG-*b*-PDLL30K	35,000	23,000	1.7
MPEG-*b*-PDLL60K	35,000	49,000	1.8
MPEG-*b*-PCL30K	65,000	25,000	2.2
MPEG-*b*-PCL60K	65,000	47,000	1.6

^a^Calculated based on monomer/MPEG ratio and MPEG molecular weight; ^b^obtained from gel permeation chromatography (GPC).

### Preparation of drug-loaded blend nanoparticles

Drug-loaded blend nanoparticles were prepared via a nanoprecipitation method. Briefly, 4 mg of indomethacin and 60 mg of copolymer blend were co-dissolved in 6 mL of acetone. The organic solution was added dropwise into 60 mL of distilled water under magnetic stirring. The nanoparticles were immediately formed after solvent extraction. The organic solvent was then evaporated at room temperature for 6 h in a fume hood. The drug-loaded blend nanoparticles with MPEG-*b*-PDLL/MPEG- *b*-PCL blend weight ratios of 100/0, 75/25, 50/50, 25/75, and 0/100 ( *w/w*) were investigated. The resultant nanoparticle suspensions were centrifuged at 15,000 rpm 4°C for 2 h. The supernatant was carefully discarded and the precipitated nanoparticles were then resuspended in a phosphate buffer solution media (0.1 M, pH 7.4). The dried drug-loaded nanoparticles were obtained by freeze-drying the precipitated nanoparticles for 48 h.

### Characterization of drug-loaded blend nanoparticles

Morphology of the drug-loaded nanoparticles was determined by transmission electron microscopy (TEM) using a JEOL JEM 1230 TEM (JEOL Ltd., Tokyo, Japan). For TEM analysis, a drop of the nanoparticle suspension was placed on a formvar film coated on a copper grid. The specimen on the copper grid was not stained. The average particle sizes of the drug-loaded nanoparticles were measured from the nanoparticle suspension by light scattering analysis using a Coulter LS230 particle size analyzer (Beckman Coulter Inc., Brea, CA, USA) at 25°C. The thermal decomposition behavior of the dried nanoparticles was characterized by thermogravimetry (TG) analysis using a TA-Instrument SDT Q600 TG analyzer (TA Instruments, New Castle, DE, USA). For TG, 5–10 mg of nanoparticles was heated from 50°C to 600°C at the rate of 20°C/min under a nitrogen atmosphere. Thermal transition properties of the dried nanoparticles were determined by mean of non-isothermal differential scanning calorimetry (DSC) using a Perkin-Elmer Pyris Diamond DSC (Perkin-Elmer Inc., Waltham, MA, USA). For DSC, 5–10 mg of nanoparticles was heated at the rate of 10°C/min under helium flow. The degree of crystallinity of the blend nanoparticles due to PCL crystallization was calculated according to the following equation [20],

(1) $$\text{Crystallinity of blend nanoparticles }\left(\%\right)=\left[\Delta {\text{H}}_{\text{m}}/135.44\right]\times 100$$

where ΔH_m_ is the melting enthalpy of the nanoparticles, and 135.44 J/g is the melting enthalpy for 100% crystalline PCL [21].

The theoretical drug loading content (DLC_theoretical_), actual drug loading content (DLC_actual_), and drug loading efficiency (DLE) were calculated from Equations 2, 3, and 4, respectively. The DLC_actual_ is an average value from three measurements. For the DLC_actual_ measurement, the dried drug-loaded nanoparticles were dissolved in dichloromethane. The weight of the actual entrapped drug was then determined by ultraviolet (UV)–vis spectrophotometry using a Perkin-Elmer Lambda 25 UV–vis spectrophotometer (Perkin-Elmer Inc., Waltham, MA, USA) at 319 nm and compared to a standard curve of indomethacin.

(2) $${\text{DLC}}_{\text{theoretical}}\left(\%\right)=\frac{\text{Weight of feed drug }}{\text{Weight of feed drug and copolymer}}\times 100$$

(3) $${\text{DLC}}_{\text{actual}}\left(\%\right)=\frac{\text{Weight of actual drug entrapped in nanoparticles }}{\text{Weight of drug-loaded nanoparticles}}\times 100$$

(4) $$\text{DLE }\left(\%\right)=\frac{{\text{DLC}}_{\text{actual}}}{{\text{DLC}}_{\text{theoretical}}}\times 100$$

### *In vitro* drug release

*In vitro* release of indomethacin from the blend nanoparticles was performed by dialysis bag diffusion technique. The drug-loaded nanoparticle suspension (10 mL) was placed in a dialysis bag, tied, and immersed into 100 mL of phosphate buffered saline (0.1 M, pH 7.4). The entire system was kept at 37°C under shaking at 100 rpm.

At predetermined time intervals, 5 mL aliquots of the release medium were withdrawn from the release medium, and the same volume of fresh buffer solution was added to continue the release test. The concentration of indomethacin released was monitored using an UV–vis spectrophotometer at 319 nm. According to a predetermined indomethacin concentration-UV absorbance standard curve, indomethacin concentration of the release medium was obtained. Percentage of indomethacin release was calculated based on the ratio of drug release at each release time and initial drug content in the nanoparticles. The average percentage release was calculated from the three experiments.

## Results and discussion

### Morphology and size

**Figure 1:**
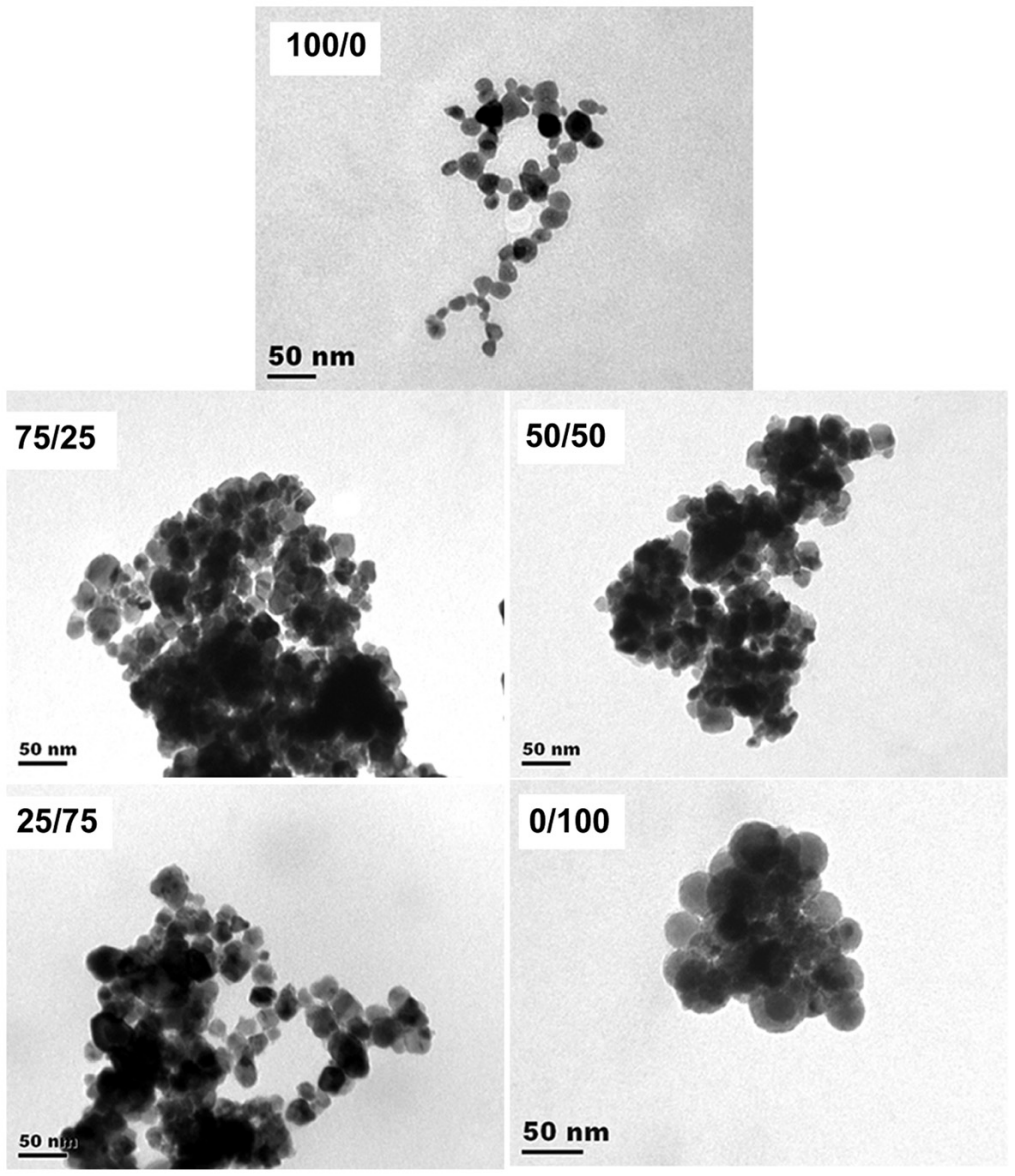
**TEM micrographs of drug-loaded nanoparticles with different MPEG-*b*-PDLL30K/MPEG-*b*-PCL30K blend weight ratios.** All bars = 50 nm.

Morphology of the blend nanoparticles was investigated by TEM, an example of which is shown in Figure 1 for the MPEG-*b*-PDLL30K/MPEG- *b*-PCL30K blend nanoparticles. It can be seen that they were nearly spherical in shape for all blend weight ratios. The blend nanoparticles of MPEG- *b*-PDLL30K/MPEG- *b*-PCL60K, MPEG- *b*-PDLL60K/MPEG- *b*-PCL30K, and MPEG- *b*-PDLL60K/MPEG- *b*-PCL60K showed similar results. The morphology results suggested that the hydrophilic MPEG blocks act as the nanoparticle shell to stabilize the nanoparticle formation [18,19]. In these nanoparticles, the hydrophobic core of the PDLL/PCL blocks is surrounded by the water-soluble polar groups of the MPEG blocks that extend into the aqueous medium to prevent nanoparticle aggregation. The polyester block length and blend weight ratio did not affect the morphology of drug-loaded blend nanoparticles. The TEM images also indicate that submicron-sized particles of copolymer blends can be prepared by the nanoprecipitation method.

**Figure 2:**
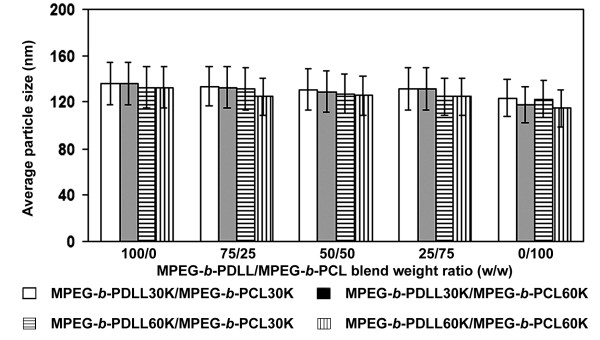
Average particle sizes of drug-loaded blend nanoparticles.

The particle size results, from light scattering analysis, are illustrated in Figure 2. They were less than 140 nm with a narrow size distribution. The average particle size slightly decreased as the MPEG-*b*-PCL blend weight ratio increased, which may be due to crystallization of the MPEG- *b*-PCL component during particle solidification that induced nanoparticle shrinkage.

### Thermal decomposition

**Figure 3:**
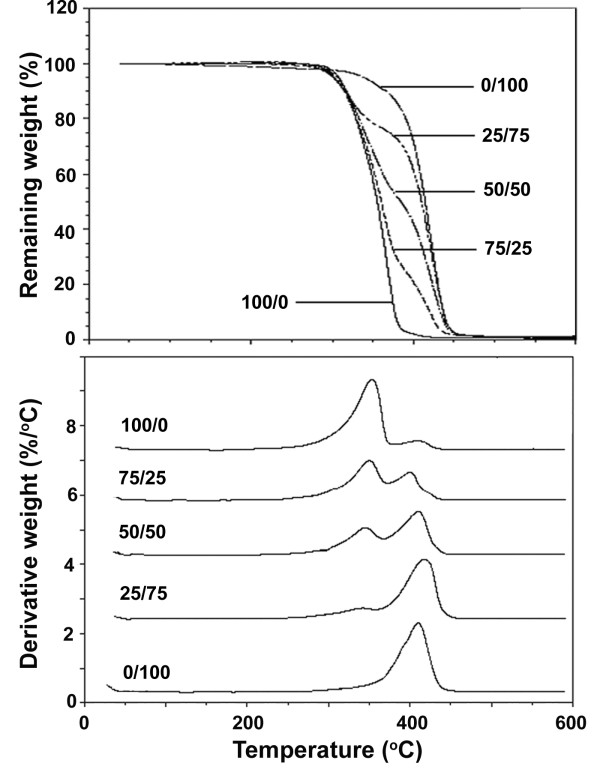
**TG (top) and DTG (bottom) thermograms of drug-loaded nanoparticles with different MPEG-*b*-PDLL30K/MPEG-*b*-PCL30K blend weight ratios.**

Thermal decomposition of the blend nanoparticles was determined by TG. The TG thermograms in Figure 3 (top) showed that the thermal resistance of the drug-loaded nanoparticles of MPEG-*b-*PCL are better than MPEG- *b*-PDLL. The blend nanoparticles, comprised of two decomposition steps, had maintained distinct thermal stability due to MPEG- *b*-PDLL decomposition, followed by degradation of the MPEG- *b*-PCL component. It was found that the TG profiles of the blend nanoparticles strongly depended upon the blend weight ratio. The amount of weight loss in the first step increased significantly when the MPEG- *b*-PDLL blend weight ratio was increased, as would be expected, since the blend nanoparticles with various blend weight ratios were successfully prepared.

Thermal decomposition can be clearly observed from differential TG (DTG) thermograms. Figure 3 (bottom) show DTG thermograms of the MPEG-*b*-PDLL30K/MPEG- *b*-PCL30K blend nanoparticles as examples. The two-step decomposition process of the blend nanoparticles is clearly illustrated. The peak temperature of the DTG thermogram is the temperature of maximum decomposition rate ( *T*_d, max_). The lower and higher *T*_d, max_ values of the DTG thermograms are attributed to MPEG-*b*-PDLL and MPEG- *b*-PCL decompositions, respectively. The T_d, max_ of MPEG-*b*-PDLL and MPEG- *b*-PCL decomposition peaks are in the range of 343°C to 354°C and 400°C to 418°C, respectively, which also indicates higher thermal stability of the MPEG- *b*-PCL matrix. It should be noted that the peak areas of the DTG thermograms strongly depend on the blend weight ratio. It can be clearly seen that the peak area of the lower temperature peak due to MPEG- *b*-PDLL decomposition decreased steadily as the MPEG- *b*-PDLL blend weight ratio decreased.

The TG and DTG thermograms of the blend nanoparticles of MPEG-*b*-PDLL30K/MPEG- *b*-PCL60K, MPEG- *b*-PDLL60K/MPEG- *b*-PCL30K, and MPEG- *b*-PDLL60K/MPEG- *b*-PCL60K changed with the blend weight ratio similar to the MPEG- *b*-PDLL30K/MPEG- *b*-PCL30K blend nanoparticles. The thermogravimetry results supported the fact that MPEG- *b*-PDLL/MPEG- *b*-PCL blend nanoparticles with different blend weight ratios were obtained.

### Thermal transition

**Figure 4:**
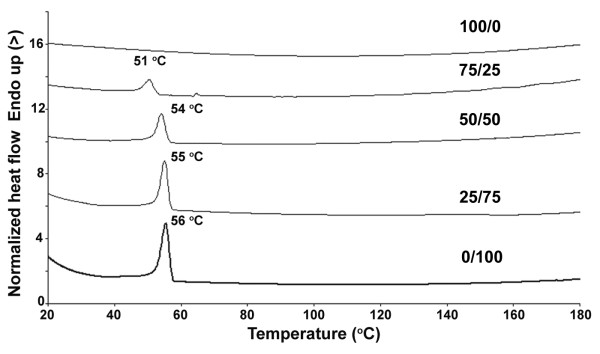
**DSC thermograms of drug-loaded nanoparticles with different MPEG-*b*-PDLL30K/MPEG-*b*-PCL30K blend weight ratios.**

The thermal transition of the blend nanoparticles was studied by DSC. The DSC thermograms of the MPEG-*b*-PDLL/MPEG- *b*-PCL blend nanoparticles are shown as an example in Figure 4. The melting peak of indomethacin is around 165°C and was not observed for all nanoparticles. This may be explained by the indomethacin molecules being incorporated into the MPEG-*b*-PDLL or MPEG- *b*-PCL in the nanoparticle matrix. The indomethacin crystallizability was then suppressed. Thus the drug was homogeneously dispersed in an amorphous form inside the nanoparticle matrix.

**Figure 5:**
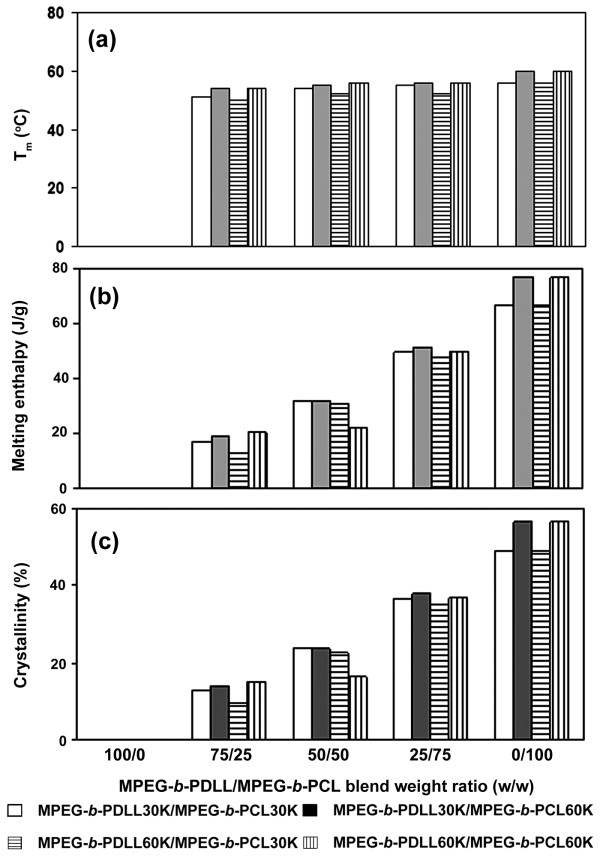
**DSC results.(a)***T*_m_, **(b)** melting enthalpy, and **(c)** crystallinity of drug-loaded blend nanoparticles.

The drug-loaded MPEG-*b*-PDLL nanoparticles were amorphous (Figure 4a). Meanwhile, the MPEG-*b*-PCL and blend nanoparticles exhibited a single melting peak due to MPEG- *b*-PCL crystalline (Figures 4b,c,d,e). The DSC results, including melting temperature (*T*_m_), melting enthalpy (ΔH_m_) and crystallinity of the nanoparticles are given in Figure 5. The *T*_m_, ΔH_m_ and crystallinity significantly decreased with the MPEG-*b*-PCL blend weight ratio. This may be explained by the MPEG- *b*-PCL crystallization being inhibited by blending with MPEG- *b*-PDLL. The results suggest that the MPEG- *b*-PDLL and MPEG- *b*-PCL can be miscible blended in the amorphous nanoparticle matrix to reduce the *T*_m_, ΔH_m_ and crystallinity of the MPEG-*b*-PCL.

### Drug loading efficiency

**Figure 6:**
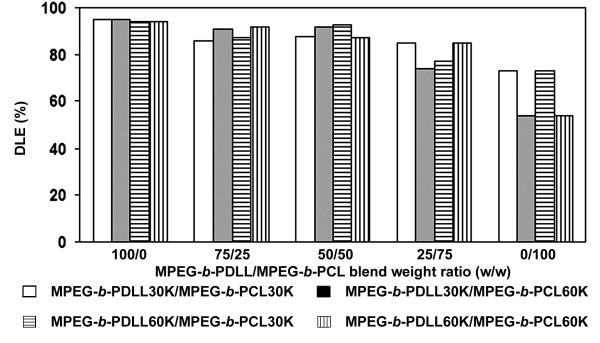
DLE of drug-loaded blend nanoparticles.

From the feed drug and copolymer blend, the theoretical drug loading contents (DLC_theoretical_) of all blend nanoparticle formulations were calculated from Equation 1, giving the result of 6.25%. The DLE of the blend nanoparticles were calculated from Equation 3 based on the DLC_theoretical_/actual drug loading content (DLC_actual_) ratios that are shown in Figure 6. The DLE was directly related to the DLC_actual_. The DLC_actual_ and DLE of MPEG-*b*-PDLL nanoparticles were higher than that of MPEG- *b*-PCL. The higher chain flexibility of MPEG- *b*-PCL is due to its lower glass transition temperature which induced faster outward drug diffusion during nanoparticle solidification. Therefore, the residue drug entrapped in the MPEG- *b*-PCL nanoparticles was less than the MPEG- *b*-PDLL nanoparticles. The DLC_actual_ and DLE of the blend nanoparticles were between MPEG-*b*-PDLL and MPEG- *b*-PCL nanoparticles.

### *In vitro* drug release

**Figure 7:**
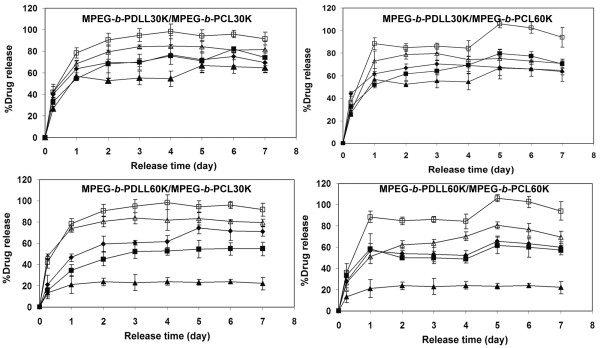
Drug release profiles from drug-loaded blend nanoparticles.

The *in vitro* drug releases from the blend nanoparticles performed in phosphate buffered saline pH 7.4 at 37°C for 7 days are illustrated in Figure 7. The drug release profiles exhibited biphasic features containing a rapid initial burst release followed by a sustaining release. The rapid initial burst release of the drug from the blend nanoparticles was probably due to the releasing of drug that was entrapped or adsorbed near to the nanoparticle surface. The initial burst release from the blend nanoparticles was found within the first 24 h of release time. After that the slow release may be due to a drug diffusion mechanism.

It can be seen that the drug release contents from the nanoparticles are in the order MPEG-*b*-PCL30K ≈ MPEG- *b*-PCL60K > MPEG- *b*-PDLL30K > MPEG- *b*-PDLL60K. The results may be explained due to the crystallinity of the MPEG- *b*-PCL component which could lead to formation of a channel structure in the nanoparticle matrix and results in the drug being easily released from the nanoparticles [10,16]. Zhang et al. [10] reported that the drug release content from the MPEG-*b*-P(CL- *co*-DLL) nanoparticles increased with the CL content. For the blend nanoparticles, with increasing MPEG- *b*-PCL blend weight ratio, the drug release increased. The higher crystallinity of the blend nanoparticles is due to the larger MPEG- *b*-PCL blend weight ratio, inducing faster drug release from the nanoparticles. The drug release results indicate that the polyester block type and blend weight ratio were important factors for controlling the drug release content from the blend nanoparticles.

## Conclusions

In the present study, drug-loaded MPEG-*b*-PDLL/MPEG- *b*-PCL blend nanoparticles with various polyester block lengths and blend weight ratios were prepared by nanoprecipitation method. They were nearly spherical in shape. The average particle size slightly decreased as the MPEG- *b*-PCL blend weight ratio increased. The thermal decomposition behaviors confirm the difference in MPEG- *b*-PDLL/MPEG- *b*-PCL blend weight ratios of the blend nanoparticles. Melting temperature and melting enthalpy depression of MPEG- *b*-PCL indicate the miscibility between MPEG- *b*-PDLL and MPEG- *b*-PCL components in the drug-loaded blend nanoparticle matrix.

The nanoparticles of MPEG-*b*-PDLL and MPEG- *b*-PCL showed the slowest and fastest drug releases, respectively. The drug release behaviors of the blend nanoparticles were between the MPEG- *b*-PCL and MPEG- *b*-PDLL nanoparticles. The drug release from the blend nanoparticles can be tailored by adjusting the PCL block length and blend weight ratio. These blend nanoparticles without any surfactants added are considered to be promising biodegradable drug carriers for sustained release of poorly water-soluble drugs.

## Competing interests

The authors declare that they have no competing interests.

## Authors’ contributions

YB developed the concept and designed the experiments. The block copolymers and drug-loaded blend nanoparticles were prepared and characterized by YB. YB and YS performed the *in vitro* drug release tests. YB wrote the manuscript. Both authors read and approved the final manuscript.
